# Protective effect of oral contraceptive against *Helicobacter pylori* infection in US adult females: NHANES 1999–2000

**DOI:** 10.1017/S0950268821000923

**Published:** 2021-04-26

**Authors:** P. Fong, Q. T. Wang

**Affiliations:** 1 School of Health Sciences and Sports, Macao Polytechnic Institute, Macao, China; 2 Key Laboratory of Drug-Targeting and Drug Delivery System of the Education Ministry and Sichuan Province, Sichuan Engineering Laboratory for Plant-Sourced Drug and Sichuan Research Center for Drug Precision Industrial Technology, West China School of Pharmacy, Sichuan University, Chengdu 610041, China

## Abstract

Recently, the antibacterial properties of oestrogen and progestogen were discovered. The aim of this study was to find the cross-sectional association between oral contraceptive use and *Helicobacter pylori* seroprevalence. Data were obtained from the US National Health and Nutrition Examination Survey (NHANES). The *H. pylori* immunoglobulin G (IgG) enzyme-linked immunosorbent assays were used to categorise participants as seropositive or seronegative. The study population included 799 female participants who had information on *H. pylori* seroprevalence and all other covariates and had not been taking any medications (except oral contraceptives). The bivariate Rao–Scott chi-square test indicated a significant association between *H. pylori* seroprevalence and contraceptive use (*P <* 0.01). The variables of race, education, poverty income ratio, smoking, and blood lead and cadmium levels were also significantly associated with *H. pylori* seroprevalence (*P <* 0.01). Multiple logistic regression analysis of the age-adjusted model revealed that contraceptive users are 65% less likely of being *H. pylori* seropositive as compared to non-contraceptive users (odds ratio (OR): 0.35, 95% confidence interval (CI): 0.18–0.68). This association is stronger with the final multivariate model (OR: 0.46, 95% CI: 0.23–0.89). *Conclusions*: This finding reveals the potential protective effect of oral contraceptives against *H. pylori* infection and serves as a foundation study for further investigations.

## Introduction

Recent studies found that the overall global prevalence of *Helicobacter pylori* infection is about 50% [1]. *H. pylori* has been a WHO-ratified class I carcinogen since 1994 and it can increase the risk of causing various types of diseases, such as gastric ulcer, gastritis, gastric adenocarcinoma, and lymphoma [2]. *H. pylori* may also decrease the acidity in the human stomach, reducing the absorption of some essential minerals and vitamins, such as iron and vitamin B_12_ [3]. Many studies have attempted to find a novel effective treatment to eradicate *H. pylori*, especially the antibiotic-resistant strains [4]. Many studies have also investigated dietary compounds that may inhibit the growth of *H. pylori*. For example, lactoferrin in cow's milk [5], sulforaphane in broccoli [6] and octyl gallate [7] as a food additive have shown their potentials in inhibiting the growth of *H. pylori*. However, reducing the global prevalence rate of *H. pylori* is still very challenging.

Many marketed drugs have shown to have therapeutic effects that have not been identified previously, including antibacterial effects. For instance, statin is a type of cholesterol-lowering drug that has demonstrated its antibacterial properties. Recently, rosuvastatin was shown to have significant antibacterial effects on both Gram-negative and Gram-positive species [8]. Thus, there is a possibility that some marketed drugs have anti-*H. pylori* properties that have not been identified. A recent study suggested that oestrogen can stimulate the production of antimicrobial peptides in the urothelial cells of women [9]. Another study found that mice feeding with oestrogen supplementation (17*β*-Oestradiol) suppressed *H. pylori*-induced gastric pathology [10]. Apart from oestrogen, another steroid hormone called progestogen was also found to have *H. pylori* bacteriolytic effects, which was stronger than that of oestradiol [11]. Oral contraceptives generally contain oestrogen and/or progestogen. This means they may affect the growth of *H. pylori* in the human gastric environment. This study aimed to study the effects of oral contraceptives on *H. pylori* prevalence using the US National Health and Nutrition Examination Survey (NHANES) dataset [12].

The NHANES is a US national programme of the Centers for Disease Control and Prevention (CDC) to study the health of Americans. The data of the NHANES were obtained by both physical examinations and interviews. The dataset has been used in many types of research, including drug repurposing (i.e. to find new medical indications for existing marketed drugs) [13]. It has also been recognised as an essential part of a systematic framework that was used to study unidentified disease–drug associations [14]. Thus, NHANES is a reputable dataset for research purposes.

## Methods

### Study population

**Fig. 1.**
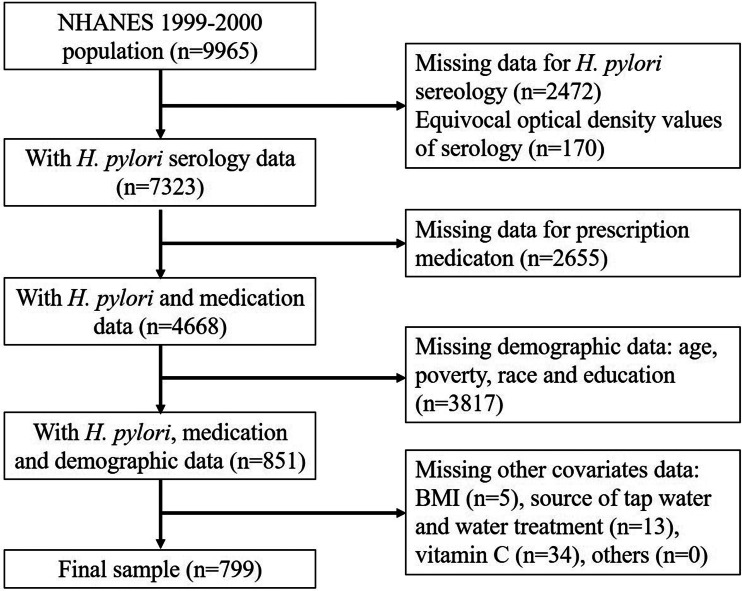
Flow chart of study populations.

Data in this study was obtained from the 1999–2000 continuous cycle of the US NHANES dataset [12]. It is a cross-sectional survey aimed at evaluating the health and nutritional status of Americans. The participants of this survey were US non-institutionalised civilians selected by the National Center for Health Statistics using a stratified and multistage probability design. The survey was approved by the US CDC, and all participants gave informed consent. The sample weights were based on the population estimated by the US Bureau of the Census [15]. The 1999–2000 cycle is the only continuous NHANES survey that contains data on *H. pylori* seropositivity. The number of participants with *H. pylori* data was 7493. After the exclusion of participants with no information on the laboratory and demographic covariates, the final sample size of this study was 799 participants (Fig. 1).

### *H. pylori* seroprevalence

*H. pylori* immunoglobulin G (IgG) enzyme-linked immunosorbent assay (ELISA) was performed on the serum samples of the 7493 participants. The assays were manufactured by Wampole Laboratories (Cranbury, NJ) and were used to detect the amount of IgG antibodies to *H. pylori* [16]. The samples were collected, stored and tested in the University of Washington under the NHANES protocol [16]. The ELISA optical density (OD) values of all the participants were ranging from 0 to 5.73. Values of >1.1 and <0.9 were considered as seropositive and seronegative, respectively, whereas 0.9–1.1 were equivocal values [17]. The participants with equivocal OD values (*N* = 170) were excluded in this study to prevent misleading statistical outcomes, and the available samples were reduced to 7323.

### Contraceptives and other drugs use assessment

The Dietary Supplements and Prescription Medication Section (DSQ) of the NHANES contains interview questions and answers regarding medication usage of the participants [18]. The questions include whether the participant had taken any medications in the past month, and if so, how long the participant had been taking the medications. The therapeutic drug class code of the medications was assigned based on the FDA's National Drug Code Directory [19]. The code for contraceptive drugs is 1040. The interviews, data collection and data processing methods were performed under the NHANES protocol [18]. Among the 15 643 participants involved in the interviews of the DSQ section, 149 had reported taking contraceptives in the past month and they were all female. As this study wants to ensure that contraceptives had enough exposure time to produce the effects on *H. pylori*, only the participants who had been taken the contraceptive for at least 90 days were involved in this study. Thus, the number of participants who took contraceptives was reduced to 131.

Medications, such as proton pump inhibitors and antacids, may affect *H. pylori* infection [20]. To exclude this potential bias, all the participants who had taken any medications (except contraceptives) in the past 90 days were excluded from this study. Among the 15 643 participants in the DSQ section, 6363 of them were not taking any medications. The total number of participants who had been taking contraceptives or no medications was 6494 (131 + 6363). Among them, only 4668 had *H. pylori* data.

### Covariate assessment

Literature suggested that *H. pylori* infection was associated with age [21], race [21], education [22], poverty [21], body mass index (BMI) [23], smoking [24], alcohol [25], caffeine [26], vitamin C [27], sources of drinking water (well or municipal) [28], home water treatment devices [29] and the amounts of lead and cadmium in blood [29]. The 1999–2000 cycle of the NHANES contains the data on these factors, and they are considered as covariates in this study. Age, race, education and poverty were categorised as listed in Table 1. The youngest participant who had been taking contraceptives was 20 years old in the NHANES dataset. BMI status was categorised into underweight (<18.5 kg/m^2^), normal or healthy weight (18.5–24.9 kg/m^2^), overweight (25.0–29.9 kg/m^2^) and obese (>30.0 kg/m^2^) according to the CDC reference values [30]. Smoking status was classified into current, previous and never smoked. Participants who had smoked at least 100 cigarettes in their entire life but not currently smoking were considered previous smokers. Alcohol users were categorised into weekly, monthly and yearly drinkers, as well as not reported, based on the interview answers in the NHANES dataset [31]. Caffeine intake (mg), vitamin C (mg), amounts of lead and cadmium in blood (μg/dl) were categorised into their approximate-weighted quartiles. The participants were asked whether home water treatment devices had been used. Water treatment devices include aerators, ceramic or charcoal filters, water softeners, reverse osmosis and Brita or other pitcher water filter [32]. The source of drinking water was categorised into water company (municipal) or public/private well according to the Housing Characteristics section of the NHANES dataset [32].

**Table 1.** Weighted characteristics of 799 participants by *H. pylori* seroprevalence, NHANES 1999–2000

Variable	Weighted participants (%)	*H. pylori* seroprevalence		*P-*values^a^
		Positive (%)	Negative (%)	
All	100	24.61	75.39	
Contraceptives				0.0095
Yes	17.5	12.8	87.2	
No	82.5	27.1	72.9	
Age (years)				0.0012
20–34	46.7	17.7	82.3	
35–50	35.8	25.7	74.3	
>50	17.5	40.8	59.2	
Race				<0.0001
Non-Hispanic White	67.5	15.1	84.9	
Non-Hispanic Black	10.5	40.2	59.8	
Other	22.0	46.3	53.7	
Education				<0.0001
Less than high school	16.8	55.1	44.9	
High school	24.5	26.3	73.7	
More than high school	58.7	15.2	84.8	
Poverty income ratio				0.0016
<2	37.2	35.5	64.5	
2–4	30.0	19.6	80.4	
>4	32.8	16.9	83.1	
BMI				0.1877
Underweight	2.5	36.0	64.0	
Normal or healthy weight	39.8	20.2	79.8	
Overweight	30.3	27.2	72.8	
Obese	27.4	27.1	72.9	
Smoking				0.0070
Current smoker	21.7	36.2	63.8	
Previous smoker	16.2	24.2	75.8	
Never smoker	62.0	20.7	79.3	
Source of tap water				0.2001
Water company	87.0	17.4	82.6	
Well water	13.0	25.7	74.3	
Water treatment device				0.0692
Yes	28.9	15.9	84.1	
No	71.1	28.2	71.8	
Blood lead (μg/dL)				0.0002
<0.9	25.2	8.4	91.6	
0.9–1.3	30.0	24.9	75.1	
1.3–2.0	21.9	31.0	69.0	
>2	22.9	36.0	64.0	
Blood cadmium (μg/dl)				0.0002
<0.3	32.8	15.4	84.6	
0.3–0.4	24.6	21.7	78.3	
0.4–0.6	19.7	28.3	71.7	
>0.6	22.9	37.7	62.3	
Vitamin C intake (mg)				0.3350
<31.3	25.0	29.2	70.8	
31.3–69.9	24.9	26.6	73.4	
69.9–147.7	25.1	22.7	77.3	
>147.7	25.0	20.0	80.0	
Caffeine intake (mg)				0.9820
<5.9	25.0	23.9	76.1	
5.9–72.9	25.0	25.3	74.7	
72.9–193.5	25.8	24.4	75.6	
>193.5	24.2	24.8	75.2	
Alcohol				0.2495
Weekly drinker	24.0	16.9	83.1	
Monthly drinker	19.7	22.6	77.4	
Yearly drinker	25.1	24.8	75.2	
Not reported	31.2	31.6	68.4	

*H. pylori*, *Helicobacter pylori*; NHANES, US National Health and Nutrition Examination Survey; BMI, body mass index.

a Rao–Scott *χ*^2^ statistics.

Among the 4668 participants who had *H. pylori* and medications data available, only 799 of them had all of the above information on covariates (Fig. 1). The final sample size was 799.

### Statistical analysis

Data were analysed using R Studio (R version 3.6.3) to evaluate the associations between the categorical variables: *H. pylori* seroprevalence, contraceptives use and the covariates. The 2-year medical examined sample weights [15] of the NHANES 1999–2000 cycle were used to account for the reference population. Bivariate associations between *H. pylori* seroprevalence and all other variables were assessed using the Rao and Scott [33] *χ*^2^ test. Multiple logistic regression analysis was conducted using the R package ‘survey’ (https://cran.r-project.org/web/packages/survey/index.html) to estimate the odds ratio (OR) and 95% confidence interval (CI) of *H. pylori* seroprevalence and contraceptive use. Covariates were adjusted to develop models using forward stepwise modelling, starting with age then poverty, race, education, BMI, smoking, source of drinking water, home water treatment device, amounts of lead and cadmium in blood, vitamin C, alcohol and caffeine.

## Results

In all, 24% of the weighted samples were *H. pylori* seropositive, and 17.5% of patients had been taking contraceptive drugs for >90 days (Table 1). Among the contraceptive drug users, 12.8% were seropositive and 87.2% were seronegative. For the non-contraceptive drug users, 27.1% and 72.9% were seropositive and seronegative, respectively (Table 1). The seropositive percentage of non-contraceptive users and contraceptive users were 27.1% and 12.8%, respectively, thus their rate ratio was 0.472 (*P* *<* 0.01). This indicates that the contraceptive users were 52.8% less likely to be *H. pylori* seropositive than those of the non-contraceptive users.

The majority of the participants involved in this study were between 20 and 34 years old (Table 1). The percentage of participants who were 50 years old or above was the lowest among all age groups (17.5%), of which 24.8% and 75.2% of them were contraceptive users and non-contraceptive users, respectively. In all, 67% of the participants were non-Hispanic Whites and >80% were at least high school graduates. Most participants had a normal or healthy weight (39.8%) or were overweight (30.3%), and only 2.5% of participants were underweight. Most of them never smoked (62%) and 25.1% of them did not drink regularly (yearly drinker). A significant proportion (31.2%) of an unknown amount of alcohol intake was observed. With regard to water-related covariates, most participants did not have a water treatment device (71.1%), and only 13% of participants had well water as their source of tap water.

**Fig. 2.**
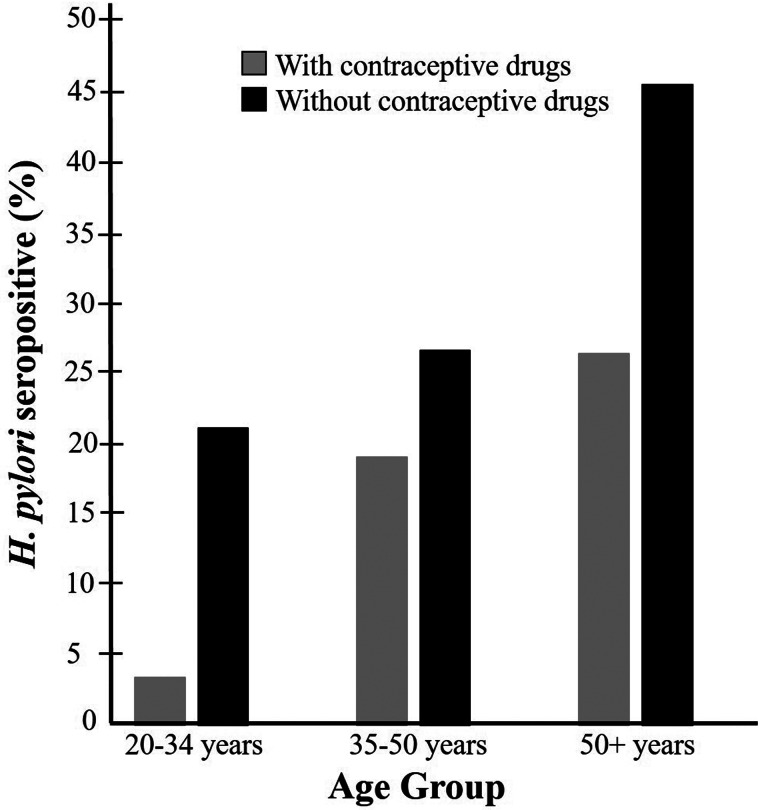
Weighted percentage of participants who were *H. pylori* seropositive by age group among those taking or not taking contraceptive drugs: NHANES 1999–2000.

The statistical bivariate test indicated a significant association between age and *H. pylori* seroprevalence (*P* *<* 0.01), and the younger age groups were less infected than the older age groups (Table 1). The greatest difference between *H. pylori* seropositivity was observed in the youngest group (20–34 years old) (Fig. 2). The variables of race, education, poverty income ratio, smoking, and blood lead and cadmium levels were also significantly associated with *H. pylori* seroprevalence (*P* *<* 0.01). Participants who were non-Hispanic White and wealthy with high educational level or with low blood lead and cadmium levels had lower *H. pylori* seroprevalence. A higher number of seropositive participants were also current smokers, as compared to the previous smoker or never smoked (*P* *<* 0.01). The percentage of underweight, overweight or obese participants with *H. pylori* seropositive status were higher than that of the participants with normal weight. However, this was not statistically significant (*P* *>* 0.05). Alcohol drinkers who drank less often tended to have a lower risk of *H. pylori* infection. Participants who consumed well water as their source of tap water tended to have higher seroprevalence than those with the water company. However, both the alcohol and source of tap water covariates were not statistically significant (*P* *>* 0.05). Vitamin C and caffeine intake also did not show significant associations with *H. pylori* seroprevalence.

The logistic regression models of each adjusted variable with contraceptive use against *H. pylori* seroprevalence are provided in Table 2. Among all these models, the race-adjusted model obtained less difference between the contraceptive use and *H. pylori* infection, the OR was 0.54, however, it was the only adjusted model with a statistically insignificant 95% CI. The range of OR among all other adjusted models was 0.38–0.51. (Table 2)

**Table 2.** Weighted associations of individual variable and contraceptive drug use against *H. pylori* seroprevalence in 799 participants, NHANES 1999–2000

Variables	Odds ratios (95% confidence intervals) of *H. pylori* seroprevalence	
	Covariates	Contraceptives (yes *vs*. no)
Age (years)		0.35 (0.18–0.68)
20–34	0.29 (0.17–0.48)	
35–50	0.44 (0.24–0.79)	
>50	Reference	
Race		0.54 (0.28–1.04)
Non-Hispanic White	0.22 (0.16–0.42)	
Non-Hispanic Black	0.76 (0.45–1.26)	
Other	Reference	
Education		0.51 (0.26–0.99)
Less than high school	Reference	
High school	0.30 (0.14–0.66)	
More than high school	0.16 (0.09–0.27)	
Poverty income ratio		0.50 (0.25–0.98)
<2	Reference	
2–4	0.46 (0.30–0.71)	
>4	0.42 (0.22–0.79)	
BMI		0.39 (0.21–0.74)
Underweight	2.21 (0.22–0.79)	
Normal or healthy weight	Reference	
Overweight	1.50 (1.09–2.08)	
Obese	1.40 (0.91–2.14)	
Smoking		0.39 (0.22–0.69)
Current smoker	2.17 (1.48–3.19)	
Previous smoker	1.41 (0.77–2.58)	
Never smoker	Reference	
Source of tap water		0.38 (0.20–0.72)
Water company	1.75 (0.85–3.60)	
Well water	Reference	
Water treatment device		0.42 (0.22–0.79)
Yes	0.50 (0.24–1.04)	
No	Reference	
Blood lead (μg/dl)		0.48 (0.27–0.86)
<0.9	Reference	
0.9–1.3	3.62 (1.58–8.27)	
1.3–2.0	4.57 (2.13–9.78)	
>2	5.65 (3.28–9.73)	
Blood cadmium (μg/dl)		0.40 (0.22–0.73)
<0.3	Reference	
0.3–0.4	1.50 (0.86–2.61)	
0.4–0.6	2.14 (1.34–3.42)	
>0.6	3.32 (2.17–5.07)	
Vitamin C intake (mg)		0.40 (0.28–0.75)
<31.3	1.61 (0.99–2.61)	
31.3–69.9	1.37 (0.79–2.38)	
69.9–147.7	0.15 (0.58–2.27)	
>147.7	Reference	
Caffeine intake (mg)		0.40 (0.21–0.73)
<5.9	0.92 (0.46–1.81)	
5.9–72.9	0.94 (0.60–1.47)	
72.9–193.5	0.93 (0.60–1.45)	
>193.5	Reference	
Alcohol		0.46 (0.24–0.89)
Weekly drinker	0.76 (0.50–1.15)	
Monthly drinker	0.72 (0.42–1.24)	
Yearly drinker	0.51 (0.29–0.91)	
Not reported	Reference	

*H. pylori*, *Helicobacter pylori*; NHANES, US National Health and Nutrition Examination Survey; BMI, body mass index.

Three multiple logistic regressions models are provided in Table 3. They were age and blood lead-adjusted, age and blood lead- and cadmium-adjusted and the final multivariate models. More multiple logistic regression analysis with all other covariates were conducted using forward stepwise modelling. These three models had the highest OR values and their 95% CI were statistically significant. The age and blood lead-adjusted model revealed that contraceptive users are 59% less likely of being *H. pylori* seropositive than non-contraceptive users (OR: 0.41, 95% CI: 0.27–0.76). This association was slightly stronger with the final multivariate model (OR: 0.46, 95% CI: 0.23–0.89).

**Table 3.** Weighted association of contraceptive drug use and covariates with *H. pylori* seroprevalence in 799 participants, NHANES 1999–2000

Variable	Odds ratios (95% confidence intervals) of *H. pylori* seroprevalence		
	Age and blood lead-adjusted	Age, blood lead and blood cadmium-adjusted	Final multivariate model
Contraceptives (yes *vs*. no)	0.41 (0.21–0.78)	0.39 (0.27–0.76)	0.46 (0.23–0.89)
Age			
20–34	0.43 (0.24–0.77)	0.47 (0.26–0.82)	0.41 (0.19–0.84)
35–50	0.50 (0.26–0.98)	0.53 (0.30–0.97)	0.56 (0.30–1.05)
>50	Reference	Reference	Reference
Blood lead (μg/dl)			
<0.9	Reference	Reference	Reference
0.9–1.3	3.18 (1.39–7.28)	2.99 (1.23–7.30)	0.38 (0.21–0.69)
1.3–2.0	4.00 (1.73–9.24)	3.53 (1.43–8.71)	1.16 (0.71–1.90)
>2	3.83 (2.25–6.51)	3.04 (1.73–5.36)	1.13 (0.59–2.14)
Blood cadmium (μg/dL)			
<0.3		Reference	
0.3–0.4		0.48 (0.32–0.74)	
0.4–0.6		0.51 (0.32–0.84)	
>0.6		2.99 (1.23–7.30)	
Education			
Less than high school			Reference
High school			0.33 (0.14–0.81)
More than high school			0.19 (0.10–0.36)
Smoking			
Current smoker			1.59 (0.95–2.68)
Previous smoker			1.38 (0.72–2.66)
Never smoker			Reference
Source of tap water			
Water company			1.76 (0.67–4.67)
Well water			Reference
Water treatment device			
Yes			0.83 (0.36–1.93)
No			Reference

*H. pylori*, *Helicobacter pylori*; NHANES, US National Health and Nutrition Examination Survey; BMI, body mass index.

## Discussion

This cross-sectional study found the association between contraceptive drug use and *H. pylori* seroprevalence using the US NHANES dataset. Various brands of contraceptive drugs exist in the US, and each brand may contain different active ingredients in different amounts and dosages. The active ingredients involved in this study were ethinyloestradiol, ethynediol, desogestrel, levonorgestrel, medroxyprogesterone, mestranol, norethindrone, norgestrel and norgestimate. They are either synthetic oestrogen or progestational hormones with different chemical structures, which affect their clinical potencies and half-lives. Oestrogen and progestogen have been shown to have *in vitro* and *in vivo* antibacterial properties on various types of bacteria, including *H. pylori* [11]. There are many existing oestrogens and progestogens and their different chemical structures affect the strength of their effects on *H. pylori*. The exact pharmacological mechanism is unknown, but they may bind to the cholesterol-binding site of bacterial cell membranes, causing bacteriostatic or even bactericidal effects [11]. Literature searches on major scientific databases were performed to find the relationship between *H. pylori* and the active ingredients that were involved in this study, and no related information could be found. Thus, we believe this is a primitive study to support the anti*-H. pylori* effects of contraceptive drugs in humans.

In this study, the results of the bivariate chi-square tests indicated associations between *H. pylori* seroprevalence and the variables of age [21], race [21], education [22], poverty [21], smoking [24] and levels of lead and cadmium in blood [29] were similar to previously published literature. Whereas, the results on BMI [23], caffeine [26], vitamin C [27], water treatment and source of drinking water [28] were different from some of the other published studies and had no association with *H. pylori* seroprevalence. The associations of these variables with *H. pylori* can be controversial. As both age and sex are identified covariates [21], the characteristics of the samples may affect their associations. For instance, Ogihara *et al*. [24] found that current smokers were at higher risk of being *H. pylori* seropositive than those who never smoked in 8837 participants older than 39 years of age. Whereas, Shinchi *et al*. [34] found no relationship between smoking and *H. pylori* infection in 566 Japanese men aged 50–55 years. Another example is caffeine intake, the study of Monno *et al*. [26] with 580 participants between 18 and 54 years of age found that coffee consumption was positively correlated with *H. pylori* status, whereas Shimamoto *et al*. [35] showed that coffee consumption was not a risk factor for *H. pylori* infection in 8013 participants. The characteristics of the participants in this study were dissimilar with the above-mentioned studies, as they were female-only and most of them were 20–50 years of age.

In this study, the 20–34 years old age group exhibited the greatest difference of seropositive rates between the contraceptive drug users and non-users (Fig. 2). This age group may have less exposure to *H. pylori* than the older groups because of their younger age. This may allow the contraceptive drug to exert their potential anti-*H. pylori* effects in an earlier stage of the infection. Also, older age participants are more likely to have other pathophysiological conditions that may make them prone to the infection, such as diabetes, systemic inflammation and other health concerns [36].

This study has several limitations. The common weaknesses of a cross-sectional study are the lack of control variables and the absence of following the participants over time. These may introduce recall bias, detection bias and information bias. A cross-sectional study may not be able to study the temporal association between the variables and outcomes. Thus, the results of this study cannot decide if the contraceptive use affected the *H. pylori* infection, or the *H. pylori* infection affected the contraceptive use.

All the participants involved in this study had been taking contraceptives drugs for at least 90 days; however, their actual length of time of taking the drugs was not considered. Bacteria are prone to develop drug resistance after long-term exposure to a drug [37]. Hence, there is a chance that *H. pylori* may react differently with different lengths of drug exposure. Analysis of the types of oestrogen and progestogen, their dosages and formulations may help to generate more meaningful results, but they were also not considered here. This is because the limited sample size restricted the possibility of categorising the various types, and the NHANES dataset did not contain information on dosages and formulations.

With regard to the smoking variables, this study only considered whether the participants were current smokers, previous smoker or never smoked and did not consider some other more precise but unavailable measurements, such as the number of cigarettes smoked per day. The water-related data obtained in the NHANES may not truly reflect the type of drinking water of the participants. For example, some participants work long shifts, and their major source of drinking water was from their workplace. The BMI variable may not be truly independent of the exposure variable and contraceptive drug use. This is because weight gain may be considered as a side effect of certain types of contraceptive drugs [38]. Also, the number of underweight participants involved in this study was too small (2.5%). This may underestimate the effects of this group of participants. With regards to alcohol consumption, a high proportion of participants did not report their alcohol drinking status (31.2%). Again, this may have affected the association between alcohol and *H. pylori* status found in this study.

Another limitation is the exclusion of other confounders shown in the literature. Examples are blood group and diet. Participants with blood groups O and A were found to have higher risks of *H. pylori* infection, whereas blood group AB was less prone to the infection [39]. Dietary compounds, such as capsaicin in chilli peppers, affect the growth of *H. pylori* [40]. The inclusion of these covariates may help to strengthen our multiple logistic regression models; however, the 1999–2000 NHANES dataset did not contain this information.

## Conclusions

This study found an association between contraceptive drug use and *H. pylori* seroprevalence. However, the effect of individual active ingredients and their corresponding dose-dependent relationship with *H. pylori* seroprevalence could not be found in this study. This was due to the sample size of each active ingredient being too small for adequate statistical power, and the dosage of each drug was not recorded in the NHANES dataset. Thus far, this study revealed the hidden protective effects of oral contraceptives against *H. pylori* infection and served as a foundation for further investigations. Future studies may investigate the pharmacological mechanism and dose-dependent relationship of the individual contraceptive ingredients on *H. pylori* prevalence. This study only considered the US population, future studies may include participants in other parts of the world with different dietary habits.

## Data Availability

All data used for analysis are freely available via online public domains (https://www.cdc.gov/nchs/nhanes/index.htm).
